# Do people with multiple sclerosis want to know their prognosis? A UK nationwide study

**DOI:** 10.1371/journal.pone.0193407

**Published:** 2018-02-28

**Authors:** Laura Dennison, Martina Brown, Sarah Kirby, Ian Galea

**Affiliations:** 1 Centre for Clinical and Community Applications of Health Psychology, Department of Psychology, University of Southampton, Southampton, United Kingdom; 2 Health Sciences, University of Southampton, Southampton, United Kingdom; 3 Clinical Neurosciences, Clinical and Experimental Sciences, Faculty of Medicine, University of Southampton, Southampton, United Kingdom; University of Liverpool, UNITED KINGDOM

## Abstract

**Background:**

Multiple sclerosis (MS) has a varied and uncertain trajectory. The recent development of analytical processing tools that draw on large longitudinal patient databases facilitates personalised long-term prognosis estimates. This has the potential to improve both shared treatment decision-making and psychological adjustment. However, there is limited research on how people with MS feel about prognosis communication and forecasting. This study investigated the prognosis communication experiences and preferences of people with MS and explored whether clinical, demographic and psychological factors are associated with prognosis information preferences.

**Methods:**

3175 UK MS Register members (59% of those with active accounts) completed an online survey containing 17 questions about prognosis communication experiences, attitudes and preferences. Participants also completed validated questionnaires measuring coping strategies, tendencies to seek out (‘monitor’) or avoid (‘blunt’) information in threatening situations, and MS risk perceptions and reported their clinical and sociodemographic characteristics. Data already held on the MS Register about participants’ quality of life, anxiety and depression symptoms and MS impact were obtained and linked to the survey data.

**Results:**

53.1% of participants had never discussed long-term prognosis with healthcare professionals. 54.2% lacked clarity about their long-term prognosis. 76% had strong preferences for receiving long-term prognosis information. 92.8% were interested in using tools that generate personalised predictions. Most participants considered prognostication useful for decision-making. Participants were more receptive to receiving prognosis information at later time-points, *versus* at diagnosis. A comprehensive set of sociodemographic, clinical and psychological variables predicted only 7.9% variance in prognosis information preferences.

**Conclusions:**

People with MS have an appetite for individualised long-term prognosis forecasting and their need for information is frequently unmet. Clinical studies deploying and evaluating interventions to support prognostication in MS are now needed. This study indicates suitable contexts and patient preferences for initial trials of long-term prognosis tools in clinical settings.

## Introduction

Multiple sclerosis (MS) is a common, chronic disease of the central nervous system which produces a variety of potentially disabling symptoms including visual disturbance, spasticity, mobility problems, speech distortions, bladder and bowel dysfunction, fatigue, pain and cognitive impairment. It is also characterised by substantial variability in disease trajectory. The vast majority of people with MS (PwMS) initially experience a relapsing-remitting phase and around 80% of these subsequently convert to a progressive phase where disability accumulates [1]. Despite established prognostic principles [2] it is challenging for neurologists to predict an individual’s short and long-term disease activity, and future disability milestones [3].

A recent advance in MS prognostication is the development and preliminary evaluation of analytic tools capable of drawing on “big data” from large longitudinal patient databases to generate individualised LTP forecast estimates [3–5]. For example, one tool currently being refined and evaluated, derives statistics from the London Ontario cohort [6] and supports 30 year forecasting by predicting time to milestones including conversion to secondary progressive disease, EDSS 6 (i.e. walking using a stick), EDSS 8 (i.e. using wheelchair) and EDSS 10 (i.e. death from MS) [3]. For each milestone, numerical and visual formulations of several confidence intervals convey the amount of certainty surrounding the estimate.

Prognostication in MS is in its infancy, but the ultimate aim is to benefit patient care. Plausibly, access to timely, personalised prognosis information might have beneficial effects. It may help shared decision-making regarding the deployment of highly effective but risky treatments in individuals predicted to have a poor prognosis, and conversely less effective but safer treatments if prognosis is predicted to be favourable. For some PwMS, prognostic forecasting could lead to reductions in emotional distress through providing a more favourable prognostic prediction than expected, or by reducing the uncertainty which PwMS describe as exceptionally challenging [7, 8] and which is consistently linked to poorer psychological wellbeing [9, 10]. However there is also the possibility of detrimental effects. For example, large confidence intervals around prognostic estimates may augment perceptions of uncertainty. Unfavourable prognosis predictions may elicit despair or fear, “nocebo” effects (where pessimistic beliefs and expectations may result in worse outcomes) or the “self-fulfilling prophecy” (where a poor prognosis leads to treatment withdrawal which then leads to poor outcome) [11].

The ethics and practices surrounding prognostication has been the subject of intensive study and debate in conditions other than MS, such as critical care [12], neurodegenerative disease [13, 14] and cancer [15]. It is fairly well accepted that it is ethically sound to offer patients prognostication if this is going to be overall advantageous, so that any possible negative effects of a poor prognosis communication (such as psychological harm) are offset by the beneficial effects of, for example, a more effective treatment. In some situations, such as uveal melanoma, the overall benefit of a prognosis-determining cytogenetic test is less clear since there is no treatment which alters this outcome. Yet, 97% of patients opt for cytogenetic prognostication, retrospective regret does not occur [16, 17] or is low [18], and there is no evidence of any harm [17]. Nearly all patients who had not been offered cytogenetic prognostication would have wanted it [16], which raises ethical concerns about physicians avoiding discussion of prognosis [19], and the physician’s “duty to prognosticate” [20], should the patients so wish.

There is limited existing research evidence about whether there is an interest in prognosis forecasting amongst PwMS. A small-scale study of a short-term (3 year forecast) prognosis prediction tool suggested that PwMS found prognosis forecasting understandable and acceptable [21]. There is also very limited research evidence about what level of understanding PwMS have about their prognosis and the nature of their communication with healthcare professionals about this. In general, research suggests that pwMS have unmet needs for a wide range of different types of information, especially around the time of diagnosis [22, 23]. However, very limited research evidence exists about what level of understanding PwMS have about their prognosis and the nature of their communication with healthcare professionals about this. German research in mild-moderately affected PwMS found that detailed prognosis counselling was rare but also found that most PwMS did not feel the need to receive more information about this [21]. However, in another German sample of more severely-affected PwMS, the majority of participants considered doctors’ communication about disease progression inadequate [24]. In a UK setting, qualitative research has revealed experiences of limited prognosis communication with healthcare professionals alongside considerable ambivalence about the prospect of receiving more detailed and personalised prognosis information [25]. Whilst PwMS recognised the potential for LTP information to be beneficial, they also considered it emotionally dangerous. Indeed, *avoiding* thinking about the future was a key strategy used by PwMS to regulate the emotional impact of living with MS [25]. Whilst emphasising the emotive nature of prognosis forecasting and highlighting some of the possible adverse effects and complexities, that study’s qualitative design and small sample size precludes generalisation to a larger population. However it generated a number of hypotheses which were testable in a large-scale quantitative study namely: (1) LTP discussion within PwMS is limited; (2) PwMS require support when prognosis is discussed; (3) some PwMS have a low preference for LTP information; (4) one can predict LTP information preferences amongst PwMS based on their clinical, sociodemographic and psychological characteristics.

In the current exploratory cross-sectional study, the primary aims were to determine the prevalence of: 1) LTP information provision 2) LTP information preference amongst PwMS. A secondary (hypothesis-generating) aim was to explore whether clinical, demographic and psychological factors are associated with, and predict LTP communication preferences.

## Method

### Design

We conducted a large, cross-sectional online survey of PwMS under approval of the University of Southampton research ethics committee (for this study) and South West-Central Bristol Research Ethics Committee (16/SW/0194, for the MS Register).

### Recruitment

We recruited PwMS through the UK MS Register (http://www.ukmsregister.org). Register members are UK residents, >18 years old, with an MS diagnosis. No additional inclusion or exclusion criteria were applied for this study. All UK MS Register members (n = 7959), were invited to participate via an email (plus one reminder), containing information about the research and links to the survey website. Consent was implied by participation. The survey was live between February and June 2015. The UK MS Society website also provided study information.

### Measures

The survey included 17 questions about LTP communication experiences, attitudes and preferences (S1 File). Items were extensively and iteratively tested for comprehensibility, acceptability and face validity with PwMS, lay members of the public, healthcare professionals, MS Register staff, and academics specialised in the construction of questionnaires. The context within which LTP is discussed or sought may be (1) primary, ie knowing one’s LTP in its own right; or (2) secondary, ie knowing one’s LTP since it might impact on decisions taken by the PwMS or their clinicians within these secondary contexts eg treatment decisions, relationships, family-planning, job matters, financial planning, drawing up a will, or end of life decisions. Different individuals will put different emphasis on these contexts depending on their personal circumstances. To minimize biasing participant responses, we planned to examine the participants’ LTP preference in its primary context, avoiding contextualization in one of these situations.

The strongest signals emerging from the recent qualitative study [25] were that coping strategies, avoidance behaviour and illness perceptions were key influences of PwMS’ LTP information preferences. Hence, these psychological features were explored using the brief COPE (coping strategies), the Miller Behavioural Scale Short Form (avoidance behaviour) and published visual analogue scales assessing perceptions of the seriousness of MS and wheelchair dependency (illness perceptions). The brief COPE [26] assesses use of 14 different coping strategies; participants responded about how they deal with life stress since their MS diagnosis. The Miller Behavioural Scale Short Form [27] (MBSS) assesses tendencies to ‘monitor’ (seek out) or ‘blunt’ (avoid) information in threatening situations; following research precedence we used only the monitoring subscale [28]. Visual analogue scales were used to assess perceptions of the seriousness (0–100) of a) MS and b) wheelchair dependency [29].

Sociodemographic data, MS type, time since onset and diagnosis was also collected. We obtained data already held on the MS Register about our participants’ anxiety and depression symptoms (HADS) [30], MS impact (MSIS-29) [31], neurological disability (EDSS [32] and EQ5D [33]); the rationale for the choice of these instruments has been detailed before [34]. Only 1.2% of respondents had recent EDSS data available so the EQ5D subjective health status item and the mobility item (dichotomised into no mobility problems vs mobility problems) were used instead as an alternative indicator of MS severity/impairment (recent data was available for 88.2% of respondents). The references provided for each tool describe their psychometric properties and scoring.

### Analysis

Data analysis was conducted using SPSS v22. The survey items about prognosis preferences and experiences were first examined using descriptive statistics. Next, we conducted univariate analyses using chi-square and point-biserial correlations to explore clinical, demographic and psychological characteristics related to the respondent’s current LTP information preference (dichotomised into *higher* (want to know a lot, want to know a little) *versus lower* (unsure, don’t want to know)). All factors showing at least a trend (p<.10) towards association with LTP information preference were subsequently entered into a logistic regression model. In order to assess whether psychological factors accounted for variance in information preferences after controlling for clinical and demographic variables, a hierarchical model was chosen. Demographic factors were entered into block 1, clinical factors were entered into block 2, and psychological variables were entered into the final block.

Recent HADS and MSIS data were available for only 44.6% and 43.3% of participants respectively. Therefore, although these variables were included in the univariate analyses, they were not included in the regression model due to the considerable respective reduction in sample size (from n=2381 to n=950).

## Results

### Sample characteristics

3175 people participated. This represents a response rate of 59% of the 5381 MS Register members who had ‘active’ accounts (i.e. had accessed or updated their data or responded to research requests within the last year), or 40% of total members (i.e. including dormant accounts). Table 1 shows participant demographic and clinical characteristics. Most participants were female (71.4%) and White British (93.4%). Age ranged from 19–82 with a mean of 52.5 years. Time since diagnosis ranged from within the last year to 54 years (mean = 12.1) and time since symptom onset ranged from within the last year to 62 years (mean = 17.3). 66.9% of participants had relapsing-remitting MS, 16.2% primary progressive, 8.0% secondary progressive. 8.3% were uncertain of their MS type.

**Table 1:** Participant characteristics.

Variable		Mean (SD) & range or N (%^a^)	N (responders^b^)
Socio-demographic variables	Age (years)	52.5 (11.1), 19–82	3134
	Gender		3168
	*Female*	2263 (71.4)	
	*Male*	905 (28.6)	
	Education		3115
	*Primary or Secondary*	665 (21.3)	
	*Occupational/diploma*	1008 (32.4)	
	*University*: *undergraduate or postgraduate*	1202 (38.6)	
	*Other*	240 (7.7)	
	Living arrangements		3069
	*With spouse/partner*	2333 (76.0)	
	*Not with spouse/partner*	736 (24.0)	
	Ethnicity		3096
	*White*: *British*	2893 (93.4)	
	*White*: *Other*	142 (4.6)	
	*All Other (Non-White)*	61 (2.0)	
	Employment status		3111
	*Employed/self-employed (full or part time)*	1285(41.3)	
	*Not working due to sickness or disability*	748 (24.0)	
	*Retired*	754 (24.2)	
	*Other*	324 (10.4)	
Clinical variables	Time since MS diagnosis (years)	12.1 (9.02), <1–54	3018
	Time since MS symptom onset (years)	17.3 (11.40), <1–62	3055

^a^ % is calculated as % of respondents who provided data for the specific item.

^b^ Number of respondents is different for each question due to missing data.

These characteristics make the current study sample largely comparable to the 7959 PwMS who were members of the MS Register at the time of study (72.2% female, mean age of 47.8 years, mean 11.1 years from diagnosis and 16.3 years from symptom onset, 65.3% relapsing-remitting MS, 14.5% primary progressive MS, 8.1% secondary progressive, 12.4% uncertain MS type or data unavailable).

### Prognosis communication experiences

Table 2 summarises data on prognosis communication experiences. 53.1% reported they had never discussed their LTP during neurology appointments. Of those who had discussed LTP, 46.4% reported that they had broached the topic themselves, and 47.0% had the topic raised by a neurologist. Less commonly other healthcare professionals or family members raised the topic. A significant proportion (13.5%) felt that the information communicated had been inconsistent between healthcare professionals, with the most frequently identified source of inconsistency being between different neurologists (53.5%) or different healthcare professionals (45.1%).

**Table 2:** Prognosis communication experiences and preferences.

Survey Item [n responders^a^]	Response options	N (%)^a^
Discussion of LTP in neurology appointment [3095]	Yes	1543 (46.9%)
	No	1642 (53.1%)
Who initiated LTP discussion^b^^,^^c^ [1444–1453]	Patient	672 (46.4%)
	Neurologist	682 (47.0%)
	MS nurse	302 (20.8%)
	GP	88 (6.1%)
	Other healthcare professional	27 (1.9%)
	Family member/friend	113 (7.8%)
	Don’t know/can’t remember	56 (3.9%)
LTP message consistency^b^ [1064]	Similar	920 (86.5%)
	Different	144 (13.5%)
Source of LTP message inconsistency^b^^,^^c^^,^^d^ [144]	Neurologists	77 (53.5%)
	GPs	42 (29.2%)
	MS specialist nurses	18 (12.5%)
	Different healthcare professionals	65 (45.1%)
Clarity about LTP [2766]	No idea	1324 (47.9%)
	Very rough idea	42 (1.5%)
	Rough idea	133 (4.8%)
	Accurate idea	651 (23.5%)
	Very accurate idea	616 (22.3%)
Frequency of thinking about prognosis [2761]	Daily	446 (16.2%)
	Weekly	691 (25.0%)
	Monthly	614 (22.2%)
	Once a year	213 (7.7%)
	Rarely	699 (25.3%)
	Never	98 (3.5%)
Who is LTP discussed with (non-healthcare professionals)^c^ [3172–3174]	Partner or spouse	1498 (47.2%)
	Parents	398 (12.5%)
	Children	363 (11.4%)
	Other family members	366 (11.5%)
	Friends	800 (25.2%)
	Colleagues at work	180 (5.7%)
	Employer	114 (3.6%)
	No-one	945 (29.8%)
Preference for LTP information: current [2762]	Want to know a lot	1182 (42.8%)
	Want to know a little	917 (33.2%)
	Not sure	337 (12.2%)
	Do not want to know	326 (11.8%)
Preference for LTP information: in future [2751]	Want to know a lot	1409 (51.2%)
	Want to know a little	772 (28.1%)
	Not sure	350 (12.7%)
	Do not want to know	220 (8.0%)
Preference for LTP information: at diagnosis [2761]	Want to know a lot	1210 (43.8%)
	Want to know a little	683 (24.7%)
	Not sure	356 (12.9%)
	Do not want to know	512 (18.5%)
Understanding LTP is useful for decisions about^c^ [2580–2741]	Treatment	1952 (71.2%)
	Relationships	955 (35.2%)
	Family planning	716 (27.8%)
	Job matters	1346 (51.3%)
	Financial planning	2107 (77.8%)
	Drawing up a will	1621 (60.2%)
	End of life medical decisions	2131 (78.3%)
Want to be informed about LTP tool availability [3055]	Yes	2707 (88.6%)
	No	348 (11.4%)
Acceptable timings of LTP tool use^c^ [3163–3173]	At diagnosis	810 (25.5%)
	Weeks/months post-diagnosis	1010 (31.8%)
	At time of treatment decision	1489 (47.0%)
	At time of life decision	1375 (43.4%)
	Other times	491 (15.5%)
	Never	228 (7.2%)
Acceptable settings for LTP tool use^c^^,^^e^ [2933–2934]	Independently, alone	1244 (42.4%)
	Independently, with S/O^f^	1088 (37.1%)
	With neurologist	822 (28.0%)
	With neurologist and S/O^f^	1368 (46.6%)
	With MS nurse	834 (28.4%)
	With MS nurse and S/O^f^	1165 (39.7%)
Desirable prognostic estimates^c^^,^^e^ [2395]	If/when need stick	1059 (36.1%)
	If/when need wheelchair	1621 (55.2%)
	If/when convert to SP^g^	1445 (76.7%)
	Life expectancy	1717 (58.5%)
Most trusted sources for LTP information (N and % participants ranking each source in the top 3 out of the 7 options) [2481–2753]	Neurologist	2147 (78.0%)
	Neurologist + tool	2087 (77.9%)
	Nurse + tool	1571 (61.5%)
	Nurse	1178 (44.9%)
	Tool	903 (33.7%)
	Other PwMS + tool	337 (13.6%)
	Other PwMS	394 (15.2%)
Endorsement of public availability of LTP tool e.g. on web [2943]	Yes	1076 (36.6%)
	No	795 (27.0%)
	Not sure	1072 (36.4%)

^a^ Number of responders is different for each question due to missing data. % is calculated as % of respondents who provided data for the specific item.

^b^ Question applicable only to participants who answered “yes” to discussion of LTP.

^c^ Response option was “Tick all that apply” therefore % will not add up to 100 and there may be different n responders for each response option.

^d^ Question applicable only to participants who answered “different” to LTP message consistency.

^e^ Applicable only to participants who did not answer “never” to acceptable timings of tool use.

^f^ S/O = significant other (friend or family member).

^g^ Response option applicable only to participants with RRMS.

There was considerable variability in whether participants felt clear about their personal LTP; 45.8% claimed to have an accurate or a very accurate understanding yet 47.9% reported they had no idea. Variability also existed in the frequency with which participants reported thinking about their LTP with many thinking about it daily or weekly (41.2%), 22.2% monthly and 33% rarely or annually. Only 3.5% claimed to never think about it. Many participants reported talking about LTP to partners/spouses (47.2%) and friends (25.2%) yet a substantial proportion did not discuss LTP with anybody (29.8%).

### Prognosis information preferences

Most participants wanted to know their LTP ‘a little’ or ‘a lot’ (68.5% at diagnosis, 76.0% at the time of survey, 79.3% in the future) but a substantial minority did not want to know (18.5% at diagnosis, 11.8% at the time of survey, 8% in the future) or were unsure (12.9% at diagnosis, 12.2% at the time of survey, 12.7% in the future). Most participants felt that LTP information would assist decision-making, particularly about treatment (71.2%), finances (77.8%) and end-of-life care (78.3%).

### Attitudes towards prognosis forecasting tools

Participants were then asked about their attitude towards a possible future electronic tool which would deliver an individualised LTP estimate, together with a measure of certainty around this estimate, after matching the individual to a subset of patients from a large database. This is the only information they were given ie they were not shown any such tool. The vast majority of participants (88.6%) stated that they would like to be informed about the availability of such a tool during neurology appointments. Participants showed interest in using an electronic prognosis prediction tool at various time points including when making treatment (47.0%) or life (43.4%) decisions, and in the weeks and months following diagnosis (31.8%). Fewer felt they would have wanted it at diagnosis (25.5%) and 7.2% stated they would never want to use it.

There was a strong association between preferences for LTP information and interest in using a tool (χ^2^ (1) = 443.20, p<.001); virtually all (98.9%) individuals with high current LTP information preferences indicated a desire to use a tool at some point (rather than “never”). Also, 75.2% of those who identified themselves as having low information preferences nonetheless indicated interest in using the tool at some point.

Participants endorsed a range of settings as acceptable for tool use including with either a neurologist (28.0%) or an MS specialist nurse (28.4%). More participants indicated they found these settings acceptable if accompanied by a significant other (46.6% and 39.7% respectively). Some participants also considered it acceptable to use a prognosis tool independently at home with (37.1%), and without (42.4%) a family member or friend present. There was interest in using the tool to predict a range of MS outcomes including necessity of a walking stick (36.1%), a wheelchair (55.2%), progression to SPMS (76.7% of the responders for whom this was relevant i.e. those with RRMS), and life expectancy (58.5%). Opinion was divided regarding whether the tool should be publically available (36.6% yes, 27.0% no, 36.4% unsure).

### Factors related to prognosis preferences

Table 3 shows associations between demographic, clinical and psychological variables and LTP information preferences. Univariate analyses suggested that participants were significantly more likely to have higher LTP information preferences if they were male and younger. Clinically, they were more likely to have RRMS, know their MS type, have shorter time since diagnosis and symptom onset, and lower (worse) subjective health status. Psychologically, higher LTP information preference was associated with higher anxiety, higher monitoring tendencies, and more use of five different coping strategies: planning, instrumental support, distraction, self-blame, and venting. All effect sizes were small [35].

**Table 3:** Relationships between demographic, clinical and psychological variables and current LTP information.

	Current LTP information preference (Dichotomised: Higher/Lower)		
	Test statistic & df	Effect size^§^	p
**Sociodemographic variables**			
Age		r = -0.058	0.002*
Gender	χ^2^ (1) = 10.584	V = 0.062	0.001*
**Clinical Variables**			
MS type	χ^2^ (3) = 21.86	V = 0.051	0.001*
Time since symptom onset		r = -0.080	<0.001*
Time since diagnosis		r = -0.087	0.001*
Subjective health status (EQ5DVAS)		r = -0.051	0.011*
Mobility (EQ5D mobility)	χ^2^ (1) = 0.206	V = 0.012	0.650
MS Impact (MSIS)		r = 0.016	0.590
**Psychological variables**			
Anxiety (HADS)		r = 0.067	0.020*
Depression (HADS)		r = 0.043	0.140
Monitoring style (MMBS)		r = 0.156	<0.001*
COPE active		r = 0.038	0.050
COPE planning		r = 0.121	<0.001*
COPE acceptance^a^	χ^2^ (1) = 0.114	V = 0.007	0.736
COPE denial^a^	χ^2^ (1) = 0.190	V = 0.008	0.663
COPE instrumental support		r = 0.062	0.001*
COPE emotional support		r = -0.002	0.899
COPE humour		r = 0.025	0.188
COPE substance use^a^	χ^2^ (1) = 3.834	V = 0.038	0.050
COPE behavioural disengagement^a^	χ^2^ (1) = 0.722	V = 0.016	0.395
COPE distraction		r = 0.040	0.038*
COPE positive reframing		r = 0.026	0.175
COPE self-blame^a^	χ^2^ (1) = 12.172	V = 0.068	<0.001*
COPE religion^a^	χ^2^ (1) = 0.685	V = 0.016	0.408
COPE venting^a^	χ^2^ (1) = 5.750	V = 0.046	0.016*
Perceived severity of MS VAS^b^	χ^2^ (1) = 0.274	V = 0.010	0.601
Perceived severity of wheelchair VAS^b^	χ^2^ (1) = 0.105	V = 0.006	0.746

^**§**^ Effect sizes: r = correlation coefficient, V = Cramer’s V.

* p<0.05.

^a^ Variable was non-normally distributed and therefore recoded into a dichotomy representing high/low use of each strategy (cut-off used was score of 4 or higher).

^b^ Variable was non-normally distributed and therefore recoded into a dichotomy representing low-medium perceived severity and higher perceived severity (cut-off used was 0.60 or higher).

Table 4 reports results from logistic regression analyses to predict LTP information preferences. Both block 1 (clinical variables) and 2 (clinical and demographic variables) were significant, explaining 1.1% and 2.5% of the variance in outcome respectively. At block 3 (clinical, demographic and psychological variables) the model was significant, explaining 7.9% of the variance and correctly classifying 77.3% of cases. The significant unique predictors were male gender, knowing MS type (compared to not knowing), higher monitoring style, and higher use of planning as a coping strategy.

**Table 4:** Logistic regression results showing significant predictors of LTP information preferences.

	B	SE	Wald	Odds Ratio	95% confidence interval
**Predictors of LTP information preferences**					
Gender (male)	-0.41	0.12	11.61*	0.66	[0.52, 0.84]
Knowing MS type	-0.52	0.15	11.74*	0.59	[0.44, 0.80]
Monitoring style	0.21	0.03	43.30*	1.23	[1.15, 1.31]
COPE planning	0.15	0.03	18.66*	1.16	[1.08, 1.24]

* = *p* <0.001.

Model χ^2^ = 127.40, *p* <0.001; Nagelkerke R^2^ = 0.079.

## Discussion

### Key findings

This large survey of PwMS in the UK breaks new ground regarding experiences and preferences regarding LTP communication. Two fundamental findings were that around half of participants claimed to have *never* discussed their LTP with their neurology team and around half claimed to have ‘no idea’ about their LTP. These results are potentially of some significance. Emotional distress is common in MS [36] and uncertainty appears to promote it [9]. Deficiencies in information and understanding also raise practical and ethical issues: without the best available information about the predicted course of their own disease, can PwMS really be fully active participants in treatment decisions?

Explanations for the apparent lack of LTP communication cannot be gained from the current study. Previous qualitative research suggests that PwMS perceive that neurologists offer little LTP information upon diagnosis and subsequently PwMS lack opportunities and courage to ask, partly because thinking about the future is threatening [25]. Little is known about the attitudes and experiences of healthcare professionals regarding LTP-related communication with PwMS; this warrants exploration in future research.

Another important new insight from this study is that many PwMS describe a strong desire for LTP information. Many PwMS claim to think about LTP regularly and LTP information is deemed useful for decision-making about treatment and broader life issues. Importantly however, this study also highlighted how a significant minority of PwMS have little or no appetite for LTP information and do not feel it is valuable to them; this group should not be overlooked. Our survey design did not allow us to gain a clear insight into the reasons behind the lack of interest in personalised prognosis information. However, previous qualitative research suggests that a) PwMS employ coping strategies that involve focussing on the present and these run counter to prognosis information-seeking and b) PwMS may be averse to gaining prognosis information for fear that negative predictions would cause demoralisation [25]. Skepticism towards the predictive accuracy of forecasting from healthcare professionals and/or prognosis software tools may also be relevant here. The study did not give participants detailed information about the likely accuracy of attempts to forecast prognosis, an issue that continues to be investigated and debated. It is unclear if and how participants’ (mis)perceptions about the accuracy of neurologist and/or tool-based forecasting may have influenced study findings regarding interest and disinterest in LTP information.

The concept of a tool delivering individualized LTP estimates was demonstrated to be of interest to a large proportion of study participants. It was perceived to be useful at various time-points and helpful for decision-making. Interestingly, participants were less positive about using the tool independently from a healthcare professional and the prospect of its public online availability. This hints at concerns about accessing and interpreting important and emotive information without the input of a healthcare professional. This aligns with qualitative findings where PwMS emphasised the simultaneous need for expertise and emotional support when receiving prognosis predictions [25].

### Predicting individual differences

Although a variety of sociodemographic, clinical and psychological factors were associated with participants’ LTP information preference, regression analysis using these variables as predictors explained very limited variance in information preference. Our very large sample size evidently permitted the detection of statistically significant but small effects that are actually of limited practical or clinical significance.

It was noteworthy that more variance in LTP information was explained by psychological factors than by clinical factors. Plausibly, this indicates that a PwMS’s mindset has more influence on LTP information preferences than their clinical profile. Alternatively, other clinical factors (e.g. EDSS, number, frequency and severity of relapses, progression pace, disease-modifying treatments), not measured within this study may be more important. These factors, as well as other psychological factors (e.g. illness perceptions, intolerance of uncertainty, personality traits) could be explored in further studies to improve understanding of what drives individual differences in LTP information preferences.

### Study strengths and limitations

Conducting research through the UK MS Register permitted efficient recruitment of a very large sample, allowing the first systematic, large-scale investigation into experiences and preferences surrounding LTP communication. However, sample composition and bias require consideration. This study had a reasonable response rate (59% of active members) and our respondents were similar to overall Register members in terms of age, gender, MS type and time since diagnosis & symptom onset. However, respondents may have been unrepresentative of Register members (and PwMS more generally) in other important and unmeasured ways. Notably, people who had more interest in their prognosis may have been more likely to opt into the study, leading to an over-estimation of appetite for LTP information. Conversely, other sample composition features hint that we may have under-estimated appetite; the sample contained a large proportion of older adults and people with long disease durations, characteristics our analyses showed were associated with *lower* LTP preferences. Finally, the study was conducted in the UK, making the international generalizability of its findings limited. Further research is needed into prognosis communication practices and patient preferences in other countries.

### Clinical and research implications

Overall our findings indicate an appetite for prognosis information that appears to be unmet. Healthcare professionals may therefore wish to increase and improve their communication about LTP with PwMS. By delivering consistent estimates [3], a LTP tool would eliminate inconsistencies in LTP information experienced by a substantial proportion of PwMS. The availability of a LTP tool in the clinical setting may also serve as a prompt for clinicians to discuss LTP with PwMS during consultations. A data-driven prognostication tool might improve a clinician’s confidence in communicating about prognosis and could help structure these conversations.

In addition to showing widespread interest in tools that deliver individualized LTP estimates, the study also showed that PwMS had preferences for the circumstances in which such tools might be used. Acceptable settings for initial clinical trials appear to be: (1) in PwMS with high LTP information preferences; (2) supervised/controlled access with a clinician and a significant other present; (3) avoiding the time of diagnosis. The increasing complexity in treatment choice is increasing clinicians’ recognition for the need to temporally separate diagnosis from treatment decision, to enable informed decision-making, during which deployment of a LTP tool would be opportune.

Importantly we demonstrated that a significant minority of PwMS do not want to know more about their predicted LTP and would not want to use a prognosis tool in any situation. Our regression analysis showed that we cannot dependably predict LTP information preferences based on the sorts of information that would be readily-available to clinicians (i.e. basic clinical and sociodemographic data). Therefore, directly asking individual PwMS about their interest in receiving LTP information seems an appropriate approach to determining whether discussing LTP is desirable at that time. It would be acceptable to ask PwMS at reasonable intervals whether they would like to discuss their prognosis, since people can change their mind. In this respect, it would be important to know, in the small proportion of PwMS who had low LTP information preference, low monitoring style and COPE planning scores, whether these characteristics change with time.

Considerable work remains to be done about how best to communicate prognosis predictions and how this influences PwMS’s behaviour, decision-making and emotional wellbeing. Clinical studies of LTP communication improvement initiatives in MS, including possible use of LTP tools, are now needed.

## Supporting information

S1 File Prognosis in MS (PIMS study) questionnaire items. (PDF)
