# Supplementary material for: Do people with multiple sclerosis want to know their prognosis? A UK nationwide study
Source: PLoS One. 2018 Feb 28;13(2):e0193407. doi: 10.1371/journal.pone.0193407 (PMC5831099; doi:10.1371/journal.pone.0193407)
Supplement: S1 File — (PDF) [file pone.0193407.s001.pdf]

Supporting Information

S1 File. Prognosis in MS (PIMS study) questionnaire items.

This questionnaire asks about your views on long-term prognosis in MS.

Your participation will enable health professionals to know how you would like to approach the issue of long-term prognosis of multiple sclerosis.

In particular your views will be sought about a computer tool which is being developed to help health professionals during discussions about long-term prognosis.

First we would like to clarify what we mean by long-term prognosis. Long-term prognosis is an estimate of how you are likely to be throughout your lifetime as a result of MS

	**Question**	**Response options**	**Response format**
1	Has your long-term prognosis ever been discussed during your neurology appointments?	• Yes<br>• No	Choose one option
1a	Who brought up the topic of long-term prognosis during your appointments?	• You<br>• Neurologist<br>• Multiple Sclerosis specialist nurse<br>• GP<br>• Other health care professional<br>• Family member or friend<br>• Don’t know / can’t remember	Tick all that apply<br><br>Only shown if answer to question 1 was “yes”
1b	If you talked to different health professionals about your long term prognosis, was the message they gave you:	• Similar. All health professionals said pretty much the same thing, even if not exactly<br>• Different. Health professionals gave clearly different messages	Choose one option<br><br>Only shown if answer to question 1 was “yes”
1c	When your long term prognosis was discussed, which health professionals gave you different messages?	• Different neurologists gave different messages<br>• Different GPs gave different messages	Choose one option<br><br>Only shown if answer to

		• Different MS specialist nurses gave different messages<br>• Different health professionals gave different messages	question 1b was “different”
2	A program is being developed that estimates an individualised and consistent long-term prognosis for people with MS. It asks some basic questions about your diagnosis and then estimates long term prognosis by matching with data from other studies. It will also inform the user how good this estimate is by giving a worst and best case scenarios specific to the individual’s case.<br><br>If development of this tool was to succeed, would you like to be informed of this tool’s availability during a clinic appointment?	• Yes<br>• No	Choose one option
3	When would you like to have the prognostic tool available to you?	• At diagnosis<br>• A few weeks or months after diagnosis<br>• When you need to make a treatment decision<br>• When you need to make a life decision<br>• At other times……………… (add/write in)<br>• Never	Tick all that apply
4	In which of the following settings would you find it acceptable to use this tool to learn about your long-term prognosis?	• Use the software myself, on my own<br>• Use the software myself, accompanied by a significant other (e.g. family member, friend)<br>• With a neurologist, unaccompanied by a significant other<br>• With a neurologist and accompanied by a significant other	Tick all that apply

		• With a MS specialist nurse, unaccompanied by a significant other<br>• With a MS specialist nurse and accompanied by a significant other<br>• Never	
5	The tool can estimate the following long-term prognoses. Which of these are you likely to want to know now?	• Whether / when you will need a stick<br>• Whether / when you will need a wheelchair<br>• Whether / when your MS will convert to the secondary progressive phase<br>• Life expectancy	Tick all that apply
6	How much will you trust the following sources of information regarding your long-term prognosis?	• Computer tool<br>• Neurologist’s opinion<br>• Combination of computer tool and neurologist’s opinion<br>• MS specialist nurse’s opinion<br>• Combination of computer tool and MS specialist nurse’s opinion<br>• The opinion of other people with MS<br>• Combination of computer tool and the opinion of other people with MS	Enter a number next to the items, where 1 is most trusted, 2 is second most trusted, and so on till you rate all 7 sources
7	Should this long-term prognosis tool be publicly available, for example on the web?	• Yes<br>• No<br>• Not sure	Choose one option
8	How clear are you about your long-term prognosis?	• I have no idea at all<br>• I have a very rough idea (give or take 20 years)<br>• I have a rough idea (give or take 10 years)<br>• I have an accurate idea (give or take 5 years)<br>• I have a very accurate idea (give or take 2 years)	Choose one option
9	Please indicate how much you want to know your long-term prognosis right now:	• A lot<br>• A little<br>• I do not want to know<br>• Not sure	Choose one option

10	Please indicate how much you would have wanted to know your long-term prognosis around the time you had your diagnosis	• A lot<br>• A little<br>• I would not have wanted to know<br>• Not sure	Choose one option
11	Please indicate how much you would want to know your long-term prognosis in the future:	• A lot<br>• A little<br>• I do not think I would ever want to know<br>• Not sure	Choose one option
12	Roughly how often do you think about your long-term prognosis?	• Daily<br>• Weekly<br>• Monthly<br>• Once a year<br>• Rarely<br>• Never	Choose one option
13	Do you discuss your long-term prognosis with any of the following people	• Partner or spouse<br>• Parents<br>• Children<br>• Other family members<br>• Friends<br>• Colleagues at work<br>• Employer<br>• No-one	Tick all that apply
14	Do you think that knowing a reliable long-term prognosis will affect your current decisions about:	• Treatment<br>• Relationships<br>• Family-planning (i.e. if and when to have children):<br>• Job matters<br>• Financial planning<br>• Drawing up a will<br>• End of life decisions (e.g. instructions about medical interventions if you became very ill or disabled)	Tick all that apply
